# Supplementary material for: Does audio-visual information result in improved health-related decision-making compared with audio-only or visual-only information? Protocol for a systematic review and meta-analysis
Source: BMJ Open. 2022 Apr 29;12(4):e059599. doi: 10.1136/bmjopen-2021-059599 (PMC9058802; doi:10.1136/bmjopen-2021-059599)
Supplement: Author's manuscript [file bmjopen-2021-059599.draft_revisions.pdf]

BMJ Open is committed to open peer review. As part of this commitment we make the peer review history of every article we publish publicly available.

When an article is published we post the peer reviewers' comments and the authors' responses online. We also post the versions of the paper that were used during peer review. These are the versions that the peer review comments apply to.

The versions of the paper that follow are the versions that were submitted during the peer review process. They are not the versions of record or the final published versions. They should not be cited or distributed as the published version of this manuscript.

BMJ Open is an open access journal and the full, final, typeset and author-corrected version of record of the manuscript is available on our site with no access controls, subscription charges or pay-per-view fees (http://bmjopen.bmj.com).

If you have any questions on BMJ Open's open peer review process please email info.bmjopen@bmj.com

BMJ Open

Does audio-visual information result in improved health-related decision making compared to audio-only or visual-only information? Protocol for a systematic review & meta-analysis

Journal:	*BMJ Open*
Manuscript ID	bmjopen-2021-059599
Article Type:	Protocol
Date Submitted by the Author:	25-Nov-2021
Complete List of Authors:	Stacey, Jemaine; Nottingham Trent University<br>Atkin, Christopher; Nottingham Trent University<br>Henshaw, Helen; University of Nottingham, School of Medicine; NIHR Nottingham Hearing Biomedical Research Unit, NIHR Nottingham Hearing Biomedical Research Unit<br>Roberts, Katherine L.; Nottingham Trent University<br>Allen, Harriet; University of Nottingham, Department of Psychology<br>Badham, Stephen; Nottingham Trent University
Keywords:	Information technology < BIOTECHNOLOGY & BIOINFORMATICS, GERIATRIC MEDICINE, Information management < BIOTECHNOLOGY & BIOINFORMATICS, World Wide Web technology < BIOTECHNOLOGY & BIOINFORMATICS, STATISTICS & RESEARCH METHODS

SCHOLARONE™
Manuscripts

PROTOCOL

Does audio-visual information result in improved health-related decision making compared to audio-only or visual-only information? Protocol for a systematic review & meta-analysis

Jemaine E. Stacey[1*], Christopher Atkin[1], Helen Henshaw[2], Katherine L. Roberts[1], Harriet Allen[3] & Stephen Badham[1]

[1]NTU Psychology, Nottingham Trent University, Nottingham, UK
[2]School of Medicine, Clinical Neuroscience, Hearing Sciences, University of Nottingham, Nottingham, UK
[3]Department of Psychology, University of Nottingham, Nottingham, UK
*Correspondence to Jemaine E. Stacey, jemaine.stacey@ntu.ac.uk

Abstract

Introduction. Making health-related decisions can be difficult due to the amount and complexity of information available. Audio-visual information may improve memory for health information but whether audio-visual information can enhance health-related decisions has not been explored using quantitative methods. The objective of this systematic review is to understand how effective audio-visual information is for informing health-related decision making compared to audio-only or visual-only information. **Methods & Analysis.** Studies will be included if they include audio-visual and either audio-only or visual-only information provision and decision making in a health setting. Studies will be excluded if they are not reported in English. Databases to be searched include: Cochrane Database of Systematic Reviews; Database of Abstracts of Reviews of Effects (DARE); Cochrane Central Register of Controlled Trials (CENTRAL); Cochrane database of methodology reviews; Cochrane methodology register; and NHS Economic Evaluation Database (NHS EED), EBSCO, Ovid EMBASE, Ovid MEDLINE, PubMEd, PsychINFO, Scopus and Web of Science (Science and Social Science Citation Index). The Cochrane Risk of Bias tool (version 7) will be used to assess risk of bias in included studies. Results will be synthesised primarily using a meta-analysis, where quantitative data is not reported a narrative synthesis will be used. **Ethics and dissemination.** No ethical issues are foreseen. Data will be disseminated via academic publication and conference presentations. Findings may also be published in scientific newsletters and magazines. This review is funded by the ESRC and is registered via PROSPERO, registration number CRD42021255725.

Keywords: audio-visual; decision making; health decisions

Article Summary

Strengths and limitations of this study

- This systematic review is the first to investigate the use of audio-visual information to improve health related decisions using meta-analysis to synthesise results.
- The level of evidence for outcomes will be assessed using the Grading of Recommendations Assessment, Development and Evaluation (GRADE).
- Grey literature will not be included.

Rationale

Individuals often have to make important decisions which can affect their health such as deciding when to seek medical advice or which treatment to pursue. Yates and Patalano define a decision as ‘the selection of an action with the aim of producing satisfying outcomes’. [1] To elaborate on this, we define the decision making process as involving the reviewing and weighting of information, then

For peer review only

making a choice between two or more options to reach a conclusion. We define audio-visual information as the concurrent presentation of auditory and visual information. This includes speech (in person or video recorded) as this is how health information would normally be delivered for example during a face-to-face consultation with a doctor.

Two reviews have been conducted in the field of health information provision focusing on audio-visual information. [2,3] Van der Meulen et al [2] focused on recall of health information whilst the most common outcome variables reported in the Wofford et al review included knowledge retention, and health attitudes. [3] No review has systematically incorporated audio-visual information and decision making from a health perspective.

Van der Meulen et al [2] reviewed 10 studies which included recall of different methods of delivering health information to cancer patients. Interventions which used auditory-only information (audio tape of consultation) and visual-only information (typed letter) were included. Overall, the research showed that providing a letter alone or an audiotape alone did not enhance recall of information, compared to a control group who received no additional information. When the letter and audiotape were directly compared there was no improvement in recall. However, when patients (mean age of 62 years) were given both a letter and audiotape, recall improved compared to the letter only (audiotape only was not included as a comparator). This suggests that the presence of both audio and visual information may improve memory for health information.

In their narrative review, Wofford et al identified 26 randomised control trials in the literature that used multimedia information presented via a computer for patient education. The authors noted that the majority of interventions involved audio information and only three studies used video. [3] Only two studies used combined audio-visual information (multimedia computer programme & printed booklet) and measured decisional conflict in three domains: i) uncertainty regarding choosing between multiple options to make a decision, ii) factors which contribute to uncertainty, and (iii) perceived effectiveness of the decision making. [4,5] Overall, audio-visual information improved decisional conflict scores in all three domains.

In particular, it is important to understand how older adults use information to inform health decisions as they will likely have to make important health decisions in later life, at a time when cognitive aging is impacting decision making processes. [6,7] Making informed health decisions may also be harder for older adults who live alone for example, those who have lost a partner, as they may have to make decisions independently. [8] Furthermore, English and Carstensen [9] demonstrated that poor health reduced the age-related positivity bias (where older adults prioritise positive information over negative information to a greater extent than young adults), during health-related decision making. This indicated that older adults may find health-related decisions particularly difficult at times when those decisions are most crucial. Recent evidence suggests audio-visual information, compared to auditory only or visual only information may disproportionately benefit cognition for older adults relative to young adults. [10,11] Therefore this review will aim to evaluate if audio-visual information may be an effective approach to improving health outcomes for older adults.

It is important to understand how older adults use information to inform health decisions as they may have to make important health decisions in later life. Making informed health decisions may be quite challenging due to the large amount of complex information available and may also be harder for older adults who live alone, for example; those who have lost a partner, as they may have to make decisions independently. [8]

As the decision-making literature is multidisciplinary, we are interested in synthesising the available evidence using meta-analysis in order to better understand how audio-visual information might improve health-related decision making. We hypothesise that audio-visual information would benefit all patients but that it may be particularly useful for older adults who might have audio or visual deficits and/or an increased number of health-related decisions to make.

Objectives

The current review aims to establish whether providing health information in dual-modality (audio-visual) can improve the speed, confidence, or quality of health-related decisions, compared to single-modality (audio-only or visual-only) information. The objective is: To understand how effective audio-visual information is for informing health-related decision making compared to audio-only or visual-only information.

Methods and analysis

Eligibility criteria

Peer-reviewed articles will be included, as well as ongoing research where datasets are available (e.g. via clinicaltrials.gov or the Open Science Framework; OSF). The Participant Intervention Comparator Outcome (PICO) framework will be used to identify papers. Only articles published in English will be considered given time constraints and lack of funds for translation. There will be no date restrictions placed on the literature searches.

Studies will be screened for inclusion of audio-visual stimuli, and audio-only and/or visual-only stimuli. Audio-only stimuli can include spoken speech, for example; telephone consultations or audio recordings. Visual-only stimuli can include pictures, animations, and written text. The audio-visual stimuli can include videos, television, or a combination of the audio-only and visual only stimuli listed. The study can include audio-visual information only or a comparison between audio-visual information and information presented in one of the formats listed above. For decision making, studies could include three distinct stages, a pre-decision phase where information is given to participants, the decision making stage where the information is considered, weighted, or a problem solving task is completed. Finally a clear choice must be made between two or more options. Decision making must be measured by a minimum of one (or more) of the following: speed of decision making, confidence of decision or quality of decision.

Participants

Research which includes adults aged 18 and above will be included. If other age groups are included within studies, but data can be separated, those studies will be eligible for inclusion.

Intervention/interest

Audio-visual health information

Comparator

Audio-only/visual-only health information

Outcomes

Health-related decision making must be measured by one or all of the following: speed of decision making, confidence of decision or quality of decision.

Subgroup analyses: If data allow, we will conduct separate meta-analyses for younger vs. older adults.

Information sources

Databases to be searched: (Cochrane Database of Systematic Reviews; Database of Abstracts of Reviews of Effects (DARE); Cochrane Central Register of Controlled Trials (CENTRAL); Cochrane database of methodology reviews; Cochrane methodology register; and NHS Economic Evaluation

For peer review only

Database (NHS EED)), EBSCO, Ovid EMBASE, Ovid MEDLINE, PubMEd, PsychINFO, Scopus and Web of Science (Science and Social Science Citation Index).

Search Strategy (OVID)

#	Searches
1	exp Decision Making/
2	exp Educational Technology/
3	health*.mp.
4	audio-visual*.mp.
5	Multimedia*.mp.
6	2 or 4 or 5
7	1 and 3 and 6

For peer review only

Article selection process
The review team will be Jemaine Stacey (JS), Christopher Atkin (CA), Helen Henshaw (HH) and Stephen Badham (SB). Two reviewers (JS & CA) will independently screen the titles and abstracts, and full-texts of retrieved studies against the inclusion/exclusion criteria. If there is any disagreement between the two reviewers, a third reviewer will be involved to reach a decision. If not enough information is provided in the titles and abstracts to know if it should be included, the full-texts will be screened by both reviewers, and a third reviewer will be involved if there is any disagreement.

Data extraction process
JS will be responsible for the creation of the data extraction form. The data extraction process will be subject to piloting by both reviewers. The data from each study will be extracted separately by JS and CA and the compared. A third reviewer will be involved if there is any disagreement.

Data items
The data extracted will include the aim, comparator, study design, setting, conflicts of interest, demographic information about the population, randomisation process, effect sizes reported for the outcomes: effectiveness of decision making, and knowledge of health information, any missing outcome data and the selection of the reported result. The authors will be contacted via email if sufficient detail is not reported and if a response is not received this will be noted. If data are only reported via figures then WebPlotDigitizer (http:// arohatgi.info/ WebPlotDigitizer/ app/) will be used to extract the data from figures. A third reviewer will be involved if there is any disagreement between reviewers regarding data extracted from plots.

Study risk of bias assessment
The reviewers JS and CA will assess risk of bias for each paper identified by using the Cochrane Risk of Bias tool (version 7) which allows a rating to be assigned of either low risk, unclear or high risk of bias. https://methods.cochrane.org/risk-bias-2

Effect measures
We will express the size of the difference in decision making for audio-visual health information and a control (audio-only or visual-only) comparison in terms of the standardized mean difference (SMD) for domains measured using different outcome measures, or the mean difference (MD) for domains measured using the same outcome measures. The calculation of the effect size will use the pooled standard deviation (between groups), or the standard deviation of the differences (within group). We will also report the 95% confidence interval (CI) for each SMD and MD. An effect size greater than 0 indicates that a larger treatment effect was observed in the treatment group relative to the control group.

Synthesis methods

For eligible studies, where sufficient data can be extracted, we will conduct random-effects meta-analyses of the SMDs or MDs, as we anticipate significant heterogeneity across interventions between studies. For each meta-analysis, we will report a summary effect size estimate in terms of the SMD or MD together with its 95% CI. Heterogeneity across studies will be examined using the I^2 statistic and its significance tested using a χ^2 test. If effect sizes are not reported, they will be calculated from standard deviations and ANOVA or t tests. A narrative synthesis will be used for studies which cannot be meta-analysed including the study design, sample size, type of multimedia information used, findings (improvements for single modality and dual-modality health information).

Where insufficient data are available for meta-analysis, study findings will be described using a narrative review. [12]

Reporting bias assessment

Funnel plots will be used to assess reporting bias and the Egger et al test will be used to assess funnel plot asymmetry.

Certainty assessment

The level of evidence for results will be assessed for each outcome using the Grading of Recommendations Assessment, Development and Evaluation (GRADE) approach which allows evidence to be rated as either low, moderate or high.

Ethics and Dissemination

This systematic review and meta-analysis does not raise any ethical issues. Results will be disseminated via scientific peer-reviewed journal articles, scientific magazines, and conference presentations.

Patient and Public involvement

Patients or the public were not involved in the creation of this protocol as it relates to secondary data.

Summary

This protocol describes the steps for a systematic review and meta-analysis to determine how effective audio-visual information is for informing health-related decision making compared to audio-only or visual-only information. This review will be the first to assess the effectiveness of audio-visual information vs. audio- or visual-only in relation to health decisions using a meta-analytic approach. The review will establish the most effective modality for delivering health information for health-related decision making. The results of the review could be used to inform specific interventions for adults to help improve their health decision making.

Word count: 1989

Acknowledgements

Thanks to Sharon Potter for guidance on the systematic review process.

Author Contributions

JS, HH, KR, HA, CA & SB all contributed to the idea for the systematic review. JS & HH led on the development of the systematic review protocol. JS, HH & CA drafted the eligibility criteria and study selection. HH, KR, HA & SB provided feedback on the manuscript and approved the final draft.

Data statement

For peer review only

No datasets were generated or analysed for this protocol.

Funding

This work was funded by the Economic and social Research Council (ESRC) grant number ES/V000071/1 and Open Access funding was provided by NTU Psychology, Nottingham Trent University, Nottingham, UK.

Competing interests

None declared

References

1. Yates, J.F., & Patalano, A.L. (1999). Decision making and aging. In D.C. Park & R.W. Morrell (Eds.), *Processing of medical information in aging patients* (pp. 31–54). Mahwah, NJ: Erlbaum.
2. Van der Meulen, N., Jansen, J., Van Dulmen, S., Bensing, J., Van Weert, J. (2008) Interventions to improve recall of medical information in cancer patients: a systematic review of the literature. *Psychooncology,* 17, 857–68.
3. Wofford, J L. Smith, E D. Miller D P. (2005). The multimedia computer for office-based patient education: a systematic review. *Patient Education and Counselling,* 59,148–157.
4. Murray, E., Davis, H. Tai S. S., Coulter, A., Gray, A. & Haines, A. (2001a) Randomised controlled trial of an interactive multimedia decision aid on benign prostatic hypertrophy in primary care. *British Medical Journal*, 323:493–6.
5. Murray, E., Davis, H. Tai S. S., Coulter, A., Gray, A. & Haines, A. (2001b). Randomised controlled trial of an interactive multimedia decision aid on hormone replacement therapy in primary care. *British Medical Journal*, 323:490–3.
6. Brand M., Markowitsch H. J. (2010). Aging and decision-making: A neurocognitive perspective. *Gerontology*, 56, 319–324.
7. Peters, E., & Bruine de Bruin, W. (2012). Aging and decision skills. In M. K. Dhami, A. Schlottmann, & M. Waldmann (Eds.), *Judgment and decision making as a skill: Learning, development, and evolution* (Vol. 5, pp. 113-1139). Cambridge, United Kingdom: Cambridge University Press.
8. Peters, E., Finucane, M. L., MacGregor, D. G., & Slovic, P. (2000). The bearable lightness of aging: Judgment and decision processes in older adults. *The aging mind: Opportunities in cognitive research*, 144-165.
9. English, T., & Carstensen, L. L. (2015). Does positivity operate when the stakes are high? Health status and decision making among older adults. *Psychology and Aging, 30*(2), 348–355.
10. de Dieuleveult, A. L., Siemonsma, P. C., van Erp, J. B., & Brouwer, A. M. (2017). Effects of aging in multisensory integration: a systematic review. *Frontiers in aging neuroscience*, *9*, 80.
11. Freiherr, J., Lundström, J. N., Habel, U., & Reetz, K. (2013). Multisensory integration mechanisms during aging. *Frontiers in human neuroscience*, *7*, 863.
12. Egger, M., Smith, G. D., Schneider, M., & Minder, C. (1997). Bias in meta-analysis detected by a simple, graphical test. *British Medical Journal*, *315*(7109), 629-634.

For peer review only

BMJ Open

Does audio-visual information result in improved health-related decision making compared to audio-only or visual-only information? Protocol for a systematic review & meta-analysis

Journal:	*BMJ Open*
Manuscript ID	bmjopen-2021-059599.R1
Article Type:	Protocol
Date Submitted by the Author:	07-Mar-2022
Complete List of Authors:	Stacey, Jemaine; Nottingham Trent University<br>Atkin, Christopher; Nottingham Trent University<br>Henshaw, Helen; University of Nottingham, School of Medicine; NIHR Nottingham Hearing Biomedical Research Unit, NIHR Nottingham Hearing Biomedical Research Unit<br>Roberts, Katherine L.; Nottingham Trent University<br>Allen, Harriet; University of Nottingham, Department of Psychology<br>Justice, Lucy; Nottingham Trent University<br>Badham, Stephen; Nottingham Trent University
**Primary Subject Heading**:	Health services research
Secondary Subject Heading:	Geriatric medicine, Communication
Keywords:	Information technology < BIOTECHNOLOGY & BIOINFORMATICS, GERIATRIC MEDICINE, Information management < BIOTECHNOLOGY & BIOINFORMATICS, World Wide Web technology < BIOTECHNOLOGY & BIOINFORMATICS, STATISTICS & RESEARCH METHODS

SCHOLARONE™
Manuscripts

PROTOCOL

Does audio-visual information result in improved health-related decision making compared to audio-only or visual-only information? Protocol for a systematic review and meta-analysis

Jemaine E. Stacey[1*], Christopher Atkin[1], Helen Henshaw[2], Katherine L. Roberts[1], Harriet A. Allen[3] Lucy V. Justice[1] & Stephen Badham[1]

[1]NTU Psychology, Nottingham Trent University, 50 Shakespeare St, Nottingham, NG1 4FQ, UK
[2]School of Medicine, Clinical Neuroscience, Hearing Sciences, University of Nottingham, Nottingham, UK
[3]Department of Psychology, University of Nottingham, Nottingham, UK
*Correspondence to Jemaine E. Stacey, jemaine.stacey@ntu.ac.uk

For peer review only

Abstract

Introduction. Making health-related decisions can be difficult due to the amount and complexity of information available. Audio-visual information may improve memory for health information but whether audio-visual information can enhance health-related decisions has not been explored using quantitative methods. The objective of this systematic review is to understand how effective audio-visual information is for informing health-related decision making compared to audio-only or visual-only information. **Methods and Analysis.** RCTs will be included if they include audio-visual and either audio-only or visual-only information provision and decision making in a health setting. Studies will be excluded if they are not reported in English. Twelve databases will be searched including: Ovid MEDLINE, PubMEd, and PsychINFO. The Cochrane Risk of Bias tool (version 7) will be used to assess risk of bias in included RCTs. Results will be synthesised primarily using a meta-analysis, where quantitative data is not reported a narrative synthesis will be used. **Ethics and dissemination.** No ethical issues are foreseen. Data will be disseminated via academic publication and conference presentations. Findings may also be published in scientific newsletters and magazines. This review is funded by the ESRC and is registered via PROSPERO, registration number CRD42021255725.

Keywords: audio-visual; decision making; health decisions

Strengths and limitations of this study

- This systematic review is the first to investigate the use of audio-visual information to improve health related decisions using meta-analysis to synthesise results.
- The level of evidence for outcomes will be assessed using the Grading of Recommendations Assessment, Development and Evaluation (GRADE).
- This protocol was developed using the PRISMA-P checklist.
- Grey literature will be included.

Rationale

Individuals often have to make important decisions which can affect their health such as deciding when to seek medical advice or which treatment to pursue. Yates and Patalano define a decision as 'the selection of an action with the aim of producing satisfying outcomes'. [1] To elaborate on this, we define the decision making process as involving the reviewing and weighting of information, then making a choice between two or more options to reach a conclusion. A related measure of interest is decisional conflict Decisional conflict which is defined by LeBlanc et al. as 'uncertainty about which course of action to take when choice among competing options involves risk, regret, or challenge to personal life values'. [2] We define audio-visual information as the concurrent presentation of auditory and visual information. This includes speech (in person or video recorded) as this is how

health information would normally be delivered for example during a face-to-face consultation with a doctor.

Two reviews have been conducted in the field of health information provision focusing on audio-visual information. [3,4] Van der Meulen et al. [3] focused on recall of health information whilst the most common outcome variables reported in the Wofford et al. review included knowledge retention, and health attitudes. [4] No review has systematically incorporated audio-visual information and decision making from a health perspective.

Van der Meulen et al. [3] reviewed 10 studies which included recall of different methods of delivering health information to cancer patients. Interventions which used auditory-only information (audio tape of consultation) and visual-only information (typed letter) were included. Overall, the research showed that providing a letter alone or an audiotape alone did not enhance recall of information, compared to a control group who received no additional information. When the letter and audiotape were directly compared there was no improvement in recall. However, when patients (mean age of 62 years) were given both a letter and audiotape, recall improved compared to the letter only (audiotape only was not included as a comparator). This suggests that the presence of both audio and visual information may improve memory for health information.

In their narrative review, Wofford et al. identified 26 randomised control trials in the literature that used multimedia information presented via a computer for patient education. The authors noted that the majority of interventions involved audio information and only three studies used video. [4] Only two studies used combined audio-visual information (multimedia computer programme and printed booklet) and measured decisional conflict. Decisional conflict was measured in three domains: i) uncertainty regarding choosing between multiple options to make a decision, ii) factors which contribute to uncertainty, and (iii) perceived effectiveness of the decision making. [5,6] Overall, audio-visual information improved decisional conflict scores in all three domains.

In particular, it is important to understand how older adults use information to inform health decisions as they will likely have to make important health decisions in later life, at a time when cognitive aging is impacting decision making processes. [7,8] Making informed health decisions may also be harder for older adults who live alone for example, those who have lost a partner, as they may have to make decisions independently. [9] Furthermore, English and Carstensen [10] demonstrated that poor health reduced the age-related positivity bias (where older adults prioritise positive information over negative information to a greater extent than young adults), during health-related decision making. This indicated that older adults may find health-related decisions particularly difficult at times when those decisions are most crucial. Recent evidence suggests audio-visual information, compared to auditory only or visual only information may disproportionately benefit cognition for older adults relative to young adults. [11,12] Therefore this review will aim to evaluate if audio-visual information may be an effective approach to improving health outcomes for older adults.

As the decision-making literature is multidisciplinary, we are interested in synthesising the available evidence using meta-analysis in order to better understand how audio-visual information might improve health-related decision making. We hypothesise that audio-visual information will benefit all patients compared to audio only or visual only information, and audio-visual information may be particularly useful for older adults who might have auditory or visual deficits and/or an increased number of health-related decisions to make.

Objectives

The current review aims to establish whether providing health information in dual-modality (audio-visual) can improve the speed, confidence, or quality of health-related decisions, compared to single-modality (audio-only or visual-only) information. The objective is: To understand how effective audio-visual information is for informing health-related decision making compared to audio-only or visual-only information.

For peer review only - http://bmjopen.bmj.com/site/about/guidelines.xhtml

Methods and analysis

This protocol was prepared in accordance with the Preferred Reporting Items for Systematic Review and Meta-Analysis Protocols (PRISMA-P) checklist.

Eligibility criteria

Peer-reviewed articles will be included, as well as ongoing research where datasets are available (e.g. via clinicaltrials.gov, opengrey.eu or the Open Science Framework; OSF). The Participant Intervention Comparator Outcome (PICO) framework will be used to identify papers. Only articles published in English will be considered given time constraints and lack of funds for translation. There will be no date restrictions placed on the literature searches. Study designs will include randomised controlled trials (RCTs).

Studies will be screened for inclusion of audio-visual stimuli, and audio-only and/or visual-only stimuli. Audio-only stimuli can include spoken speech, for example; telephone consultations or audio recordings. Visual-only stimuli can include pictures, animations, and written text. The audio-visual stimuli can include videos, television, or a combination of the audio-only and visual only stimuli listed. The study can include audio-visual information only or a comparison between audio-visual information and information presented in one of the formats listed above. For decision making, studies could include three distinct stages, a pre-decision phase where information is given to participants, the decision making stage where the information is considered, weighted, or a problem solving task is completed. Finally a clear choice must be made between two or more options. Decision making must be measured by a minimum of one (or more) of the following: decisional conflict, confidence in decision or quality of decision. Decisions must be made by an individual for themselves not on behalf of others.

Participants

Research which includes adults aged 18 and above will be included. If other age groups are included within studies, but data can be separated, those studies will be eligible for inclusion.

Intervention/interest

Audio-visual health information

Comparator

Audio-only/visual-only health information

Outcomes

Health-related decision making must be measured by one or all of the following: speed of decision making, confidence of decision or quality of decision.

Subgroup analyses: If data allow, we will conduct separate meta-analyses for younger vs. older adults or include age group as a subgroup analysis.

Information sources

Databases to be searched: Cochrane Database of Systematic Reviews; Database of Abstracts of Reviews of Effects (DARE); Cochrane Central Register of Controlled Trials (CENTRAL); Cochrane database of methodology reviews; Cochrane methodology register; EBSCO, Ovid EMBASE, Ovid

For peer review only

MEDLINE, PubMEd, PsychINFO, Scopus, and Web of Science (Science and Social Science Citation Index).

Articles identified from the searches will be managed in Refworks to deduplicate citations. Abstracts will then be uploaded to Covidence for screening. Preliminary searches to test search terms were carried out in October 2021 the full search will be conducted in March 2022, and the searches will be repeated before publication, estimated submission date: December 2022.

The following search strategies were developed with input from a specialist librarian.

Search Strategies for different databases

#	Ovid: MEDLINE, EMBASE, PsychINFO<br>exp = explode MeSH, .mp = search titles, abstracts & keywords
1	exp Decision Making/
2	exp Educational Programs/
3	health*.mp.
4	audio-visual*.mp.
5	Multimedia*.mp.
6	2 or 4 or 5
7	1 and 3 and 6

Article selection process

The review team will be Jemaine Stacey (JS), Christopher Atkin (CA), and Helen Henshaw (HH). Two reviewers (JS & CA) will independently screen the titles and abstracts of retrieved studies against the inclusion/exclusion criteria. If there is any disagreement between the two reviewers, a third reviewer will be involved to reach a decision. If there is not enough information is provided in the titles and abstracts to know if it should be included, the full-texts will be screened. In the next step of article selection, the full-texts will be screened by both reviewers, and a third reviewer will be involved if there is any disagreement. The references lists of included articles will be checked for relevant research, and the citations of included research articles will be entered into Google Scholar to check for any new or relevant data. We have included the Cochrane Database of Systematic Reviews so that the reference lists of review articles can be checked to ensure relevant articles are captured.

Data extraction process

JS will be responsible for the creation of the data extraction form. The data extraction process will be subject to piloting by both reviewers. The data from each study will be extracted separately by JS and CA and the compared. A third reviewer will be involved if there is any disagreement.

Data items

The data extracted will include the aim, comparator, study design (e.g. RCT), the decision making task (e.g. choosing a treatment option), setting, conflicts of interest, demographic information about the population, randomisation process, effect sizes reported for the primary outcomes: decision making measures (e.g. decisional conflict/effectiveness/confidence/ N of people who made a decision), and secondary outcome: knowledge of health information, any missing outcome data and the selection of the reported result. The authors will be contacted via email if sufficient detail is not reported and if a response is not received this will be noted. If data are only reported via figures then WebPlotDigitizer (http:// arohatgi.info/ WebPlotDigitizer/ app/) will be used to extract the data from figures. A third reviewer will be involved if there is any disagreement between reviewers regarding data extracted from plots.

Study risk of bias assessment

For peer review only

The reviewers JS and CA will assess risk of bias at the study level for each RCT identified using the Cochrane Risk of Bias tool (version 7) which allows a rating to be assigned of either low risk, unclear or high risk of bias. https://methods.cochrane.org/risk-bias-2.

Effect measures

We will express the size of the difference in decision making for audio-visual health information and a control (audio-only or visual-only) comparison in terms of the standardized mean difference (SMD; cohen's *d*). We will use the SMD as we expect that decision making will be measured using different outcome measures. The calculation of the effect size will use the pooled standard deviation (between groups), or the standard deviation of the differences (within group). We will also report the 95% confidence interval (CI) for each SMD. SMDs will be corrected using Hedges' *g* which corrects for bias caused by small sample sizes. [13] An effect size greater than 0 indicates that a larger effect was observed for the intervention group compared to the control group. A positive effect size would represent an improvement in health-decisions, for example; a reduction in decisional conflict or increased confidence in decision making.

Synthesis methods

For eligible studies, if effect sizes are not reported, they will be calculated from means and standard deviations, or from ANOVA or t tests. Where sufficient data can be extracted, we will conduct random-effects meta-analyses of the SMDs, as we anticipate significant heterogeneity across interventions between studies. The studies will be weighted using the inverse variance method. Heterogeneity across studies will be examined using the I^2 statistic and significance tested using a χ^2 test. If heterogeneity is found a prediction interval for the true intervention effects will be calculated. [14] We will also check for outliers and conduct an influencer analysis to see if removing any outliers/influential studies effects the I^2. If data allow, we will include a subgroup analysis of age group (e.g. young adults 18-30 years and older adults 65-80 years). We will include risk of bias as a moderator in the analysis. Analysis will be conducted in R using the Metafor package. [15,16]

A narrative synthesis will be used for studies which cannot be meta-analysed including the study design, sample size, type of multimedia information used, findings (improvements for single modality and dual-modality health information).

JS will keep an ongoing record of any changes to the protocol via PROSPERO, and any deviations from the protocol will be included in the final published version of the systematic review.

Reporting bias assessment

Funnel plots will be used to assess reporting bias and the Egger et al. [17] test will be used to assess funnel plot asymmetry.

Certainty assessment

The level of evidence for results will be assessed for each outcome using the Grading of Recommendations Assessment, Development and Evaluation (GRADE) approach which allows evidence to be rated as either low, moderate or high.

Ethics and Dissemination

This systematic review and meta-analysis does not raise any ethical issues. Results will be disseminated via scientific peer-reviewed journal articles, scientific magazines, and conference presentations.

Patient and Public involvement

Patients or the public were not involved in the creation of this protocol as it relates to secondary data.

Word count: 2249

Acknowledgements

Thanks to Sharon Potter for guidance on the systematic review process and help developing the search strategy.

Author Contributions

JS, HH, KR, HA, CA & SB all contributed to the idea for the systematic review. JS & HH led on the development of the systematic review protocol. JS, HH & CA drafted the eligibility criteria and study selection plans. LJ aided in the development of the meta-analysis plans. HH, KR, HA & SB provided feedback on the manuscript and approved the final draft of the protocol. JS is the guarantor of the review.

Data statement

No datasets were generated or analysed for this protocol.

Funding

This work was funded by the Economic and social Research Council (ESRC) grant number ES/V000071/1 and Open Access funding was provided by NTU Psychology, Nottingham Trent University, Nottingham, UK.

Competing interests

None declared

Contact information

Jemaine Stacey Jemaine.stacey@ntu.ac.uk
Christopher Atkin christopher.atkin02@ntu.ac.uk
Stephen Badham stephen.badham@ntu.ac.uk
Kate Roberts kate.roberts@ntu.ac.uk
Helen Henshaw helen.henshaw@nottingham.ac.uk
Harriet Allen h.a.allen@nottingham.ac.uk
Lucy Justice lucy.justice@ntu.ac.uk

References

1. Yates, J.F., & Patalano, A.L. (1999). Decision making and aging. In D.C. Park & R.W. Morrell (Eds.), *Processing of medical information in aging patients* (pp. 31–54). Mahwah, NJ: Erlbaum.
2. LeBlanc A, Kenny DA, O'Connor AM, Légaré F. Decisional conflict in patients and their physicians: a dyadic approach to shared decision making. *Medical Decision Making*, *29*(1), 61-8.
3. Van der Meulen, N., Jansen, J., Van Dulmen, S., Bensing, J., Van Weert, J. (2008) Interventions to improve recall of medical information in cancer patients: a systematic review of the literature. *Psychooncology, 17*, 857–68.
4. Wofford, J L. Smith, E D. Miller D P. (2005). The multimedia computer for office-based patient education: a systematic review. *Patient Education and Counselling, 59*,148–157.

For peer review only

5. Murray, E., Davis, H. Tai S. S., Coulter, A., Gray, A. & Haines, A. (2001a) Randomised controlled trial of an interactive multimedia decision aid on benign prostatic hypertrophy in primary care. *British Medical Journal*, *323*,493–6.
6. Murray, E., Davis, H. Tai S. S., Coulter, A., Gray, A. & Haines, A. (2001b). Randomised controlled trial of an interactive multimedia decision aid on hormone replacement therapy in primary care. *British Medical Journal*, *323*,490–3.
7. Brand M., Markowitsch H. J. (2010). Aging and decision-making: A neurocognitive perspective. *Gerontology*, *56*, 319–324.
8. Peters, E., & Bruine de Bruin, W. (2012). Aging and decision skills. In M. K. Dhami, A. Schlottmann, & M. Waldmann (Eds.), *Judgment and decision making as a skill: Learning, development, and evolution* (Vol. 5, pp. 113-1139). Cambridge, United Kingdom: Cambridge University Press.
9. Peters, E., Finucane, M. L., MacGregor, D. G., & Slovic, P. (2000). The bearable lightness of aging: Judgment and decision processes in older adults. *The aging mind: Opportunities in cognitive research*, 144-165.
10. English, T., & Carstensen, L. L. (2015). Does positivity operate when the stakes are high? Health status and decision making among older adults. *Psychology and Aging, 30*(2), 348–355.
11. de Dieuleveult, A. L., Siemonsma, P. C., van Erp, J. B., & Brouwer, A. M. (2017). Effects of aging in multisensory integration: a systematic review. *Frontiers in aging neuroscience*, *9*, 80.
12. Freiherr, J., Lundström, J. N., Habel, U., & Reetz, K. (2013). Multisensory integration mechanisms during aging. *Frontiers in human neuroscience*, *7*, 863.
13. Hedges, Larry V. 1981. Distribution Theory for Glass's Estimator of Effect Size and Related Estimators. *Journal of Educational Statistics, 6* (2): 107–28.
14. IntHout, J., Ioannidis, J. P., Rovers, M. M., & Goeman, J. J. (2016). Plea for routinely presenting prediction intervals in meta-analysis. *BMJ open*, *6*(7), e010247.
15. R Core Team (2020). *R: A language and environment for statistical computing.* R Foundation for Statistical Computing, Vienna, Austria
16. Viechtbauer W (2010). Conducting meta-analyses in R with the metafor package. *Journal of Statistical Software, 36*(3), 1–48.
17. Egger, M., Smith, G. D., Schneider, M., & Minder, C. (1997). Bias in meta-analysis detected by a simple, graphical test. *British Medical Journal*, *315*(7109), 629-634.

For peer review only

PRISMA-P (Preferred Reporting Items for Systematic review and Meta-Analysis Protocols) 2015 checklist: recommended items to address in a systematic review protocol*

Section and topic	Item No	Checklist item
**ADMINISTRATIVE INFORMATION**		
Title:		
Identification	1a	Identify the report as a protocol of a systematic review
Update	1b	If the protocol is for an update of a previous systematic review, identify as such
Registration	2	If registered, provide the name of the registry (such as PROSPERO) and registration number
Authors:		
Contact	3a	Provide name, institutional affiliation, e-mail address of all protocol authors; provide physical mailing address of corresponding author
Contributions	3b	Describe contributions of protocol authors and identify the guarantor of the review
Amendments	4	If the protocol represents an amendment of a previously completed or published protocol, identify as such and list changes; otherwise, state plan for documenting important protocol amendments
Support:		
Sources	5a	Indicate sources of financial or other support for the review
Sponsor	5b	Provide name for the review funder and/or sponsor
Role of sponsor or funder	5c	Describe roles of funder(s), sponsor(s), and/or institution(s), if any, in developing the protocol
**INTRODUCTION**		
Rationale	6	Describe the rationale for the review in the context of what is already known
Objectives	7	Provide an explicit statement of the question(s) the review will address with reference to participants, interventions, comparators, and outcomes (PICO)
**METHODS**		
Eligibility criteria	8	Specify the study characteristics (such as PICO, study design, setting, time frame) and report characteristics (such as years considered, language, publication status) to be used as criteria for eligibility for the review
Information sources	9	Describe all intended information sources (such as electronic databases, contact with study authors, trial registers or other grey literature sources) with planned dates of coverage
Search strategy	10	Present draft of search strategy to be used for at least one electronic database, including planned limits, such that it could be repeated
Study records:		
Data management	11a	Describe the mechanism(s) that will be used to manage records and data throughout the review

For peer review only

Selection process	11b	State the process that will be used for selecting studies (such as two independent reviewers) through each phase of the review (that is, screening, eligibility and inclusion in meta-analysis)
Data collection process	11c	Describe planned method of extracting data from reports (such as piloting forms, done independently, in duplicate), any processes for obtaining and confirming data from investigators
Data items	12	List and define all variables for which data will be sought (such as PICO items, funding sources), any pre-planned data assumptions and simplifications
Outcomes and prioritization	13	List and define all outcomes for which data will be sought, including prioritization of main and additional outcomes, with rationale
Risk of bias in individual studies	14	Describe anticipated methods for assessing risk of bias of individual studies, including whether this will be done at the outcome or study level, or both; state how this information will be used in data synthesis
Data synthesis	15a	Describe criteria under which study data will be quantitatively synthesised
	15b	If data are appropriate for quantitative synthesis, describe planned summary measures, methods of handling data and methods of combining data from studies, including any planned exploration of consistency (such as I^2, Kendall's τ)
	15c	Describe any proposed additional analyses (such as sensitivity or subgroup analyses, meta-regression)
	15d	If quantitative synthesis is not appropriate, describe the type of summary planned
Meta-bias(es)	16	Specify any planned assessment of meta-bias(es) (such as publication bias across studies, selective reporting within studies)
Confidence in cumulative evidence	17	Describe how the strength of the body of evidence will be assessed (such as GRADE)

*** It is strongly recommended that this checklist be read in conjunction with the PRISMA-P Explanation and Elaboration (cite when available) for important clarification on the items. Amendments to a review protocol should be tracked and dated. The copyright for PRISMA-P (including checklist) is held by the PRISMA-P Group and is distributed under a Creative Commons Attribution Licence 4.0.**

From: Shamseer L, Moher D, Clarke M, Ghersi D, Liberati A, Petticrew M, Shekelle P, Stewart L, PRISMA-P Group. Preferred reporting items for systematic review and meta-analysis protocols (PRISMA-P) 2015: elaboration and explanation. BMJ. 2015 Jan 2;349(jan02 1):g7647.

For peer review only

BMJ Open

Does audio-visual information result in improved health-related decision making compared to audio-only or visual-only information? Protocol for a systematic review and meta-analysis

Journal:	*BMJ Open*
Manuscript ID	bmjopen-2021-059599.R2
Article Type:	Protocol
Date Submitted by the Author:	07-Apr-2022
Complete List of Authors:	Stacey, Jemaine; Nottingham Trent University<br>Atkin, Christopher; Nottingham Trent University<br>Henshaw, Helen; University of Nottingham, School of Medicine; NIHR Nottingham Hearing Biomedical Research Unit, NIHR Nottingham Hearing Biomedical Research Unit<br>Roberts, Katherine L.; Nottingham Trent University<br>Allen, Harriet; University of Nottingham, Department of Psychology<br>Justice, Lucy; Nottingham Trent University<br>Badham, Stephen; Nottingham Trent University
**Primary Subject Heading**:	Health services research
Secondary Subject Heading:	Geriatric medicine, Communication
Keywords:	Information technology < BIOTECHNOLOGY & BIOINFORMATICS, GERIATRIC MEDICINE, Information management < BIOTECHNOLOGY & BIOINFORMATICS, World Wide Web technology < BIOTECHNOLOGY & BIOINFORMATICS, STATISTICS & RESEARCH METHODS

SCHOLARONE™
Manuscripts

PROTOCOL

Does audio-visual information result in improved health-related decision making compared to audio-only or visual-only information? Protocol for a systematic review and meta-analysis

Jemaine E. Stacey[1*], Christopher Atkin[1], Helen Henshaw[2], Katherine L. Roberts[1], Harriet A. Allen[3] Lucy V. Justice[1] & Stephen Badham[1]

[1]NTU Psychology, Nottingham Trent University, 50 Shakespeare St, Nottingham, NG1 4FQ, UK
[2]School of Medicine, Clinical Neuroscience, Hearing Sciences, University of Nottingham, Nottingham, UK
[3]Department of Psychology, University of Nottingham, Nottingham, UK
*Correspondence to Jemaine E. Stacey, jemaine.stacey@ntu.ac.uk

Abstract

Introduction. Making health-related decisions can be difficult due to the amount and complexity of information available. Audio-visual information may improve memory for health information but whether audio-visual information can enhance health-related decisions has not been explored using quantitative methods. The objective of this systematic review is to understand how effective audio-visual information is for informing health-related decision making compared to audio-only or visual-only information. **Methods and Analysis.** RCTs will be included if they include audio-visual and either audio-only or visual-only information provision and decision making in a health setting. Studies will be excluded if they are not reported in English. Twelve databases will be searched including: Ovid MEDLINE, PubMEd, and PsychINFO. The Cochrane Risk of Bias tool (version 7) will be used to assess risk of bias in included RCTs. Results will be synthesised primarily using a meta-analysis, where quantitative data is not reported a narrative synthesis will be used. **Ethics and dissemination.** No ethical issues are foreseen. Data will be disseminated via academic publication and conference presentations. Findings may also be published in scientific newsletters and magazines. This review is funded by the ESRC and is registered via PROSPERO, registration number CRD42021255725.

Keywords: audio-visual; decision making; health decisions

Strengths and limitations of this study

- This systematic review is the first to investigate the use of audio-visual information to improve health related decisions using meta-analysis to synthesise results.
- The level of evidence for outcomes will be assessed using the Grading of Recommendations Assessment, Development and Evaluation (GRADE).
- This protocol was developed using the PRISMA-P checklist.
- Grey literature will be included.
- Studies will only be included if they are published in English.

Rationale

Individuals often have to make important decisions which can affect their health such as deciding when to seek medical advice or which treatment to pursue. Yates and Patalano define a decision as 'the selection of an action with the aim of producing satisfying outcomes'. [1] To elaborate on this, we define the decision making process as involving the reviewing and weighting of information, then making a choice between two or more options to reach a conclusion. A related measure of interest is decisional conflict Decisional conflict which is defined by LeBlanc et al. as 'uncertainty about which course of action to take when choice among competing options involves risk, regret, or challenge to personal life values'. [2] We define audio-visual information as the concurrent presentation of

For peer review only

auditory and visual information. This includes speech (in person or video recorded) as this is how health information would normally be delivered for example during a face-to-face consultation with a doctor.

Two reviews have been conducted in the field of health information provision focusing on audio-visual information. [3,4] Van der Meulen et al. [3] focused on recall of health information whilst the most common outcome variables reported in the Wofford et al. review included knowledge retention, and health attitudes. [4] No review has systematically incorporated audio-visual information and decision making from a health perspective.

Van der Meulen et al. [3] reviewed 10 studies which included recall of different methods of delivering health information to cancer patients. Interventions which used auditory-only information (audio tape of consultation) and visual-only information (typed letter) were included. Overall, the research showed that providing a letter alone or an audiotape alone did not enhance recall of information, compared to a control group who received no additional information. When the letter and audiotape were directly compared there was no improvement in recall. However, when patients (mean age of 62 years) were given both a letter and audiotape, recall improved compared to the letter only (audiotape only was not included as a comparator). This suggests that the presence of both audio and visual information may improve memory for health information.

In their narrative review, Wofford et al. identified 26 randomised control trials in the literature that used multimedia information presented via a computer for patient education. The authors noted that the majority of interventions involved audio information and only three studies used video. [4] Only two studies used combined audio-visual information (multimedia computer programme and printed booklet) and measured decisional conflict. Decisional conflict was measured in three domains: i) uncertainty regarding choosing between multiple options to make a decision, ii) factors which contribute to uncertainty, and (iii) perceived effectiveness of the decision making. [5,6] Overall, audio-visual information improved decisional conflict scores in all three domains.

In particular, it is important to understand how older adults use information to inform health decisions as they will likely have to make important health decisions in later life, at a time when cognitive aging is impacting decision making processes. [7,8] Making informed health decisions may also be harder for older adults who live alone for example, those who have lost a partner, as they may have to make decisions independently. [9] Furthermore, English and Carstensen [10] demonstrated that poor health reduced the age-related positivity bias (where older adults prioritise positive information over negative information to a greater extent than young adults), during health-related decision making. This indicated that older adults may find health-related decisions particularly difficult at times when those decisions are most crucial. Recent evidence suggests audio-visual information, compared to auditory only or visual only information may disproportionately benefit cognition for older adults relative to young adults. [11,12] Therefore this review will aim to evaluate if audio-visual information may be an effective approach to improving health outcomes for older adults.

As the decision-making literature is multidisciplinary, we are interested in synthesising the available evidence using meta-analysis in order to better understand how audio-visual information might improve health-related decision making. We hypothesise that audio-visual information will benefit all patients compared to audio only or visual only information, and audio-visual information may be particularly useful for older adults who might have auditory or visual deficits and/or an increased number of health-related decisions to make.

Objectives

The current review aims to establish whether providing health information in dual-modality (audio-visual) can improve the speed, confidence, or quality of health-related decisions, compared to single-modality (audio-only or visual-only) information. The objective is: To understand how effective

audio-visual information is for informing health-related decision making compared to audio-only or visual-only information.

Methods and analysis

This protocol was prepared in accordance with the Preferred Reporting Items for Systematic Review and Meta-Analysis Protocols (PRISMA-P) checklist.

Eligibility criteria

Peer-reviewed articles will be included, as well as ongoing research where datasets are available (e.g. via clinicaltrials.gov, opengrey.eu or the Open Science Framework; OSF). The Participant Intervention Comparator Outcome (PICO) framework will be used to identify papers. Only articles published in English will be considered given time constraints and lack of funds for translation. There will be no date restrictions placed on the literature searches. Study designs will include randomised controlled trials (RCTs).

Studies will be screened for inclusion of audio-visual stimuli, and audio-only and/or visual-only stimuli. Audio-only stimuli can include spoken speech, for example; telephone consultations or audio recordings. Visual-only stimuli can include pictures, animations, and written text. The audio-visual stimuli can include videos, television, or a combination of the audio-only and visual only stimuli listed. The study can include audio-visual information only or a comparison between audio-visual information and information presented in one of the formats listed above. For decision making, studies could include three distinct stages, a pre-decision phase where information is given to participants, the decision making stage where the information is considered, weighted, or a problem solving task is completed. Finally a clear choice must be made between two or more options. Decision making must be measured by a minimum of one (or more) of the following: decisional conflict, confidence in decision or quality of decision. Decisions must be made by an individual for themselves not on behalf of others.

Participants

Research which includes adults aged 18 and above will be included. If other age groups are included within studies, but data can be separated, those studies will be eligible for inclusion.

Intervention/interest

Audio-visual health information

Comparator

Audio-only/visual-only health information

Outcomes
Health-related decision making must be measured by one or all of the following: decisional conflict, confidence in decision or quality of decision.

Subgroup analyses: If data allow, we will conduct separate meta-analyses for younger vs. older adults or include age group as a subgroup analysis.

Information sources

Databases to be searched: Cochrane Database of Systematic Reviews; Database of Abstracts of Reviews of Effects (DARE); Cochrane Central Register of Controlled Trials (CENTRAL); Cochrane

For peer review only

database of methodology reviews; Cochrane methodology register; EBSCO, Ovid EMBASE, Ovid MEDLINE, PubMEd, PsychINFO, Scopus, and Web of Science (Science and Social Science Citation Index).

Articles identified from the searches will be managed in Refworks to deduplicate citations. Abstracts will then be uploaded to Covidence for screening. Preliminary searches to test search terms were carried out in October 2021 the full search will be conducted in March 2022, and the searches will be repeated before publication, estimated submission date: December 2022.

The following search strategies were developed with input from a specialist librarian.

Search Strategies for different databases

#	Ovid: MEDLINE, EMBASE, PsychINFO<br>exp = explode MeSH, .mp = search titles, abstracts & keywords
1	exp Decision Making/
2	exp Educational Programs/
3	health*.mp.
4	audio-visual*.mp.
5	Multimedia*.mp.
6	2 or 4 or 5
7	1 and 3 and 6

Article selection process

The review team will be Jemaine Stacey (JS), Christopher Atkin (CA), and Helen Henshaw (HH). Two reviewers (JS & CA) will independently screen the titles and abstracts of retrieved studies against the inclusion/exclusion criteria. If there is any disagreement between the two reviewers, a third reviewer will be involved to reach a decision. If there is not enough information is provided in the titles and abstracts to know if it should be included, the full-texts will be screened. In the next step of article selection, the full-texts will be screened by both reviewers, and a third reviewer will be involved if there is any disagreement. The references lists of included articles will be checked for relevant research, and the citations of included research articles will be entered into Google Scholar to check for any new or relevant data. We have included the Cochrane Database of Systematic Reviews so that the reference lists of review articles can be checked to ensure relevant articles are captured.

Data extraction process

JS will be responsible for the creation of the data extraction form. The data extraction process will be subject to piloting by both reviewers. The data from each study will be extracted separately by JS and CA and the compared. A third reviewer will be involved if there is any disagreement.

Data items

The data extracted will include the aim, comparator, study design (e.g. RCT), the decision making task (e.g. choosing a treatment option), setting, conflicts of interest, demographic information about the population, randomisation process, effect sizes reported for the primary outcomes: decision making measures (e.g. decisional conflict/effectiveness/confidence/ N of people who made a decision), and secondary outcome: knowledge of health information, any missing outcome data and the selection of the reported result. The authors will be contacted via email if sufficient detail is not reported and if a response is not received this will be noted. If data are only reported via figures then WebPlotDigitizer (http:// arohatgi.info/ WebPlotDigitizer/ app/) will be used to extract the data from figures. A third reviewer will be involved if there is any disagreement between reviewers regarding data extracted from plots.

For peer review only

Study risk of bias assessment

The reviewers JS and CA will assess risk of bias at the study level for each RCT identified using the Cochrane Risk of Bias tool (version 7) which allows a rating to be assigned of either low risk, unclear or high risk of bias. https://methods.cochrane.org/risk-bias-2.

Effect measures

We will express the size of the difference in decision making for audio-visual health information and a control (audio-only or visual-only) comparison in terms of the standardized mean difference (SMD; cohen's *d*). We will use the SMD as we expect that decision making will be measured using different outcome measures. The calculation of the effect size will use the pooled standard deviation (between groups), or the standard deviation of the differences (within group). We will also report the 95% confidence interval (CI) for each SMD. SMDs will be corrected using Hedges' *g* which corrects for bias caused by small sample sizes. [13] An effect size greater than 0 indicates that a larger effect was observed for the intervention group compared to the control group. A positive effect size would represent an improvement in health-decisions, for example; a reduction in decisional conflict or increased confidence in decision making.

Synthesis methods

For eligible studies, if effect sizes are not reported, they will be calculated from means and standard deviations, or from ANOVA or t tests. Where sufficient data can be extracted, we will conduct random-effects meta-analyses of the SMDs, as we anticipate significant heterogeneity across interventions between studies. The studies will be weighted using the inverse variance method. Heterogeneity across studies will be examined using the I^2 statistic and significance tested using a χ^2 test. If heterogeneity is found a prediction interval for the true intervention effects will be calculated. [14] We will also check for outliers and conduct an influencer analysis to see if removing any outliers/influential studies effects the I^2. If data allow, we will include a subgroup analysis of age group (e.g. young adults 18-30 years and older adults 65-80 years). We will include risk of bias as a moderator in the analysis. Analysis will be conducted in R using the Metafor package. [15,16]

A narrative synthesis will be used for studies which cannot be meta-analysed including the study design, sample size, type of multimedia information used, findings (improvements for single modality and dual-modality health information).

JS will keep an ongoing record of any changes to the protocol via PROSPERO, and any deviations from the protocol will be included in the final published version of the systematic review.

Reporting bias assessment

Funnel plots will be used to assess reporting bias and the Egger et al. [17] test will be used to assess funnel plot asymmetry.

Certainty assessment

The level of evidence for results will be assessed for each outcome using the Grading of Recommendations Assessment, Development and Evaluation (GRADE) approach which allows evidence to be rated as either low, moderate or high.

Ethics and Dissemination

This systematic review and meta-analysis does not raise any ethical issues. Results will be disseminated via scientific peer-reviewed journal articles, scientific magazines, and conference presentations.

Patient and Public involvement

For peer review only

Patients or the public were not involved in the creation of this protocol as it relates to secondary data.

Word count: 2249

Acknowledgements

Thanks to Sharon Potter for guidance on the systematic review process and help developing the search strategy.

Author Contributions

JS, HH, KR, HA, CA & SB all contributed to the idea for the systematic review. JS & HH led on the development of the systematic review protocol. JS, HH & CA drafted the eligibility criteria and study selection plans. LJ aided in the development of the meta-analysis plans. HH, KR, HA & SB provided feedback on the manuscript and approved the final draft of the protocol. JS is the guarantor of the review.

Data statement

No datasets were generated or analysed for this protocol.

Funding

This work was funded by the Economic and social Research Council (ESRC) grant number ES/V000071/1 and Open Access funding was provided by NTU Psychology, Nottingham Trent University, Nottingham, UK.

Competing interests

None declared

Contact information

Jemaine Stacey Jemaine.stacey@ntu.ac.uk
Christopher Atkin christopher.atkin02@ntu.ac.uk
Stephen Badham stepen.badham@ntu.ac.uk
Kate Roberts kate.roberts@ntu.ac.uk
Helen Henshaw helen.henshaw@nottingham.ac.uk
Harriet Allen h.a.allen@nottingham.ac.uk
Lucy Justice lucy.justice@ntu.ac.uk

References

1. Yates, J.F., & Patalano, A.L. (1999). Decision making and aging. In D.C. Park & R.W. Morrell (Eds.), *Processing of medical information in aging patients* (pp. 31–54). Mahwah, NJ: Erlbaum.
2. LeBlanc A, Kenny DA, O'Connor AM, Légaré F. Decisional conflict in patients and their physicians: a dyadic approach to shared decision making. *Medical Decision Making*, *29*(1), 61-8.

3. Van der Meulen, N., Jansen, J., Van Dulmen, S., Bensing, J., Van Weert, J. (2008) Interventions to improve recall of medical information in cancer patients: a systematic review of the literature. *Psychooncology, 17*, 857–68.

For peer review only

4. Wofford, J L. Smith, E D. Miller D P. (2005). The multimedia computer for office-based patient education: a systematic review. *Patient Education and Counselling, 59*,148–157.
5. Murray, E., Davis, H. Tai S. S., Coulter, A., Gray, A. & Haines, A. (2001a) Randomised controlled trial of an interactive multimedia decision aid on benign prostatic hypertrophy in primary care. *British Medical Journal*, *323*,493–6.
6. Murray, E., Davis, H. Tai S. S., Coulter, A., Gray, A. & Haines, A. (2001b). Randomised controlled trial of an interactive multimedia decision aid on hormone replacement therapy in primary care. *British Medical Journal*, *323*,490–3.
7. Brand M., Markowitsch H. J. (2010). Aging and decision-making: A neurocognitive perspective. *Gerontology*, *56*, 319–324.
8. Peters, E., & Bruine de Bruin, W. (2012). Aging and decision skills. In M. K. Dhami, A. Schlottmann, & M. Waldmann (Eds.), *Judgment and decision making as a skill: Learning, development, and evolution* (Vol. 5, pp. 113-1139). Cambridge, United Kingdom: Cambridge University Press.
9. Peters, E., Finucane, M. L., MacGregor, D. G., & Slovic, P. (2000). The bearable lightness of aging: Judgment and decision processes in older adults. *The aging mind: Opportunities in cognitive research*, 144-165.
10. English, T., & Carstensen, L. L. (2015). Does positivity operate when the stakes are high? Health status and decision making among older adults. *Psychology and Aging, 30*(2), 348–355.
11. de Dieuleveult, A. L., Siemonsma, P. C., van Erp, J. B., & Brouwer, A. M. (2017). Effects of aging in multisensory integration: a systematic review. *Frontiers in aging neuroscience*, *9*, 80.
12. Freiherr, J., Lundström, J. N., Habel, U., & Reetz, K. (2013). Multisensory integration mechanisms during aging. *Frontiers in human neuroscience*, *7*, 863.
13. Hedges, Larry V. 1981. Distribution Theory for Glass's Estimator of Effect Size and Related Estimators. *Journal of Educational Statistics, 6* (2): 107–28.
14. IntHout, J., Ioannidis, J. P., Rovers, M. M., & Goeman, J. J. (2016). Plea for routinely presenting prediction intervals in meta-analysis. *BMJ open*, *6*(7), e010247.
15. R Core Team (2020). *R: A language and environment for statistical computing.* R Foundation for Statistical Computing, Vienna, Austria
16. Viechtbauer W (2010). Conducting meta-analyses in R with the metafor package. *Journal of Statistical Software, 36*(3), 1–48.
17. Egger, M., Smith, G. D., Schneider, M., & Minder, C. (1997). Bias in meta-analysis detected by a simple, graphical test. *British Medical Journal*, *315*(7109), 629-634.

For peer review only

PRISMA-P (Preferred Reporting Items for Systematic review and Meta-Analysis Protocols) 2015 checklist: recommended items to address in a systematic review protocol*

Section and topic	Item No	Checklist item
**ADMINISTRATIVE INFORMATION**		
Title:		
Identification	1a	Identify the report as a protocol of a systematic review see title
Update	1b	If the protocol is for an update of a previous systematic review, identify as such n/a
Registration	2	If registered, provide the name of the registry (such as PROSPERO) and registration number see final line of abstract
Authors:		
Contact	3a	Provide name, institutional affiliation, e-mail address of all protocol authors; provide physical mailing address of corresponding author see both author affiliations and 'contact information' provided on page 6 line 32
Contributions	3b	Describe contributions of protocol authors and identify the guarantor of the review see 'author contributions' page 6 line 12 onwards
Amendments	4	If the protocol represents an amendment of a previously completed or published protocol, identify as such and list changes; otherwise, state plan for documenting important protocol amendments N/A
Support:		
Sources	5a	Indicate sources of financial or other support for the review see 'funding' page 6 line 22 onwards
Sponsor	5b	Provide name for the review funder and/or sponsor see 'funding' page 6 line 22 onwards
Role of sponsor or funder	5c	Describe roles of funder(s), sponsor(s), and/or institution(s), if any, in developing the protocol see 'funding' page 6 line 22 onwards
**INTRODUCTION**		
Rationale	6	Describe the rationale for the review in the context of what is already known page 1 line 44 onwards
Objectives	7	Provide an explicit statement of the question(s) the review will address with reference to participants, interventions, comparators, and outcomes (PICO) see page 2 'Objectives' line 49 onwards AND the PICO is on page 3 lines 29-48
**METHODS**		
Eligibility criteria	8	Specify the study characteristics (such as PICO, study design, setting, time frame) and report characteristics (such as years considered, language, publication status) to be used as criteria for eligibility for the review see page 3 'eligibility criteria' lines 7-27
Information sources	9	Describe all intended information sources (such as electronic databases, contact with study authors, trial registers or other grey literature sources) with planned dates of coverage see 'information sources' page 3 lines 50-54 and page 4 lines 1-9
Search strategy	10	Present draft of search strategy to be used for at least one electronic database, including planned limits, such that it could be

For peer review only

		repeated see table on page 4 line 11
Study records:		
Data management	11a	Describe the mechanism(s) that will be used to manage records and data throughout the review page 4 lines 4-7
Selection process	11b	State the process that will be used for selecting studies (such as two independent reviewers) through each phase of the review (that is, screening, eligibility and inclusion in meta-analysis) see page 4 lines 15-18 and lines 27-29
Data collection process	11c	Describe planned method of extracting data from reports (such as piloting forms, done independently, in duplicate), any processes for obtaining and confirming data from investigators see page 4 lines 27-29
Data items	12	List and define all variables for which data will be sought (such as PICO items, funding sources), any pre-planned data assumptions and simplifications page 4 lines 31-41
Outcomes and prioritization	13	List and define all outcomes for which data will be sought, including prioritization of main and additional outcomes, with rationale page 4 lines 34-36
Risk of bias in individual studies	14	Describe anticipated methods for assessing risk of bias of individual studies, including whether this will be done at the outcome or study level, or both; state how this information will be used in data synthesis see page 5 lines 1-3
Data synthesis	15a	Describe criteria under which study data will be quantitatively synthesised see synthesis methods page 5 lines 17-27
	15b	If data are appropriate for quantitative synthesis, describe planned summary measures, methods of handling data and methods of combining data from studies, including any planned exploration of consistency (such as I^2, Kendall's τ)
	15c	Describe any proposed additional analyses (such as sensitivity or subgroup analyses, meta-regression) page 5 lines 25-27
	15d	If quantitative synthesis is not appropriate, describe the type of summary planned page 5 lines 29-31
Meta-bias(es)	16	Specify any planned assessment of meta-bias(es) (such as publication bias across studies, selective reporting within studies) see page 5 lines 38-39
Confidence in cumulative evidence	17	Describe how the strength of the body of evidence will be assessed (such as GRADE) see 5 lines 43-45

*** It is strongly recommended that this checklist be read in conjunction with the PRISMA-P Explanation and Elaboration (cite when available) for important clarification on the items. Amendments to a review protocol should be tracked and dated.** **The copyright for PRISMA-P (including checklist) is held by the PRISMA-P Group and is distributed under a Creative Commons Attribution Licence 4.0.**

From: Shamseer L, Moher D, Clarke M, Ghersi D, Liberati A, Petticrew M, Shekelle P, Stewart L, PRISMA-P Group. Preferred reporting items for systematic review and meta-analysis protocols (PRISMA-P) 2015: elaboration and explanation. BMJ. 2015 Jan 2;349(jan02 1):g7647.

For peer review only
